# Pulmonary intravascular lymphoma diagnosed by 18-fluorodeoxyglucose positron emission tomography-guided transbronchial lung biopsy in a man with long-term survival: a case report

**DOI:** 10.1186/1752-1947-5-295

**Published:** 2011-07-07

**Authors:** Tomiharu Niida, Kikuo Isoda, Koji Miyazaki, Soichiro Kanoh, Hideo Kobayashi, Ayako Kobayashi, Fumihiko Kimura, Katsumi Hayashi, Masatoshi Kusuhara, Fumitaka Ohsuzu

**Affiliations:** 1 Division of Cardiovascular Medicine, National Defense Medical College, 3-2 Namiki, Tokorozawa, Saitama 359-8513, Japan; 2 Division of Pulmonary Medicine, National Defense Medical College, 3-2 Namiki, Tokorozawa, Saitama 359-8513, Japan; 3 Division of Hematology, Department of Internal Medicine, National Defense Medical College, 3-2 Namiki, Tokorozawa, Saitama 359-8513, Japan; 4 Department of Radiology, National Defense Medical College, 3-2 Namiki, Tokorozawa, Saitama 359-8513, Japan; 5 Regional Resources Division, Shizuoka Cancer Center Research Institute, 1007 Shimonagakubo, Nagaizumi-cho, Sunto-gun, Shizuoka 411-8777, Japan

## Abstract

**Introduction:**

18-Fluorodeoxyglucose positron emission tomography can detect the pulmonary involvement of intravascular lymphoma that presents no abnormality in a computed tomography scan.

**Case presentation:**

We report the case of a 61-year-old Japanese man who had pulmonary intravascular lymphoma and no computed tomography abnormality. We were able to make an antemortem diagnosis of pulmonary intravascular lymphoma by transbronchial lung biopsy according to 18-fluorodeoxyglucose positron emission tomography findings. He is free of recurrent disease 24 months after chemotherapy.

**Conclusions:**

To the best of our knowledge, this is the first reported case of a long-term survivor of pulmonary intravascular lymphoma diagnosed by transbronchial lung biopsy under the guide of 18-fluorodeoxyglucose positron emission tomography.

## Introduction

Intravascular lymphoma (IVL), a rare subtype of extranodal diffuse large B-cell lymphoma, is characterized by malignant cells that proliferate within small vessels [1]. We recently treated a patient who had pulmonary IVL and who showed no abnormality in computed tomography (CT) scans. Gallium scintigraphy showed slightly increased opacity in both of his lungs. Conversely, 18-fluorodeoxyglucose positron emission tomography (FDG-PET) revealed strongly increased tracer levels in the lower regions of both lungs. We performed a transbronchial lung biopsy (TBLB) according to the FDG accumulation and established a histological diagnosis. In our patient, FDG-PET proved to be more useful in diagnosing IVL after other radiological modalities - high-resolution computed tomography (HRCT) and gallium scintigraphy - had failed to identify pulmonary lesions. This is a case of pulmonary IVL diagnosed by TBLB under the guide of FDG-PET findings.

## Case presentation

A 61-year-old Japanese man with a four-month history of dyspnea was referred to our cardiovascular department because of unexplained dyspnea. His symptoms included low-grade fever (37°C to 38°C), night sweats, and progressive dyspnea on exertion during the preceding month. A physical examination revealed a loud diastolic murmur in his right second intercostal space but no jugular vein distention, respiratory crackles, or leg edema. Laboratory data indicated pancytopenia, inflammation, and liver dysfunction. An examination of the blood showed a hemoglobin level of 11.5 g/dL, a hematocrit level of 33.2%, a platelet count of 8.3 × 10^4^/μL, and a white blood cell count of 5.4 × 10^3^/μL. His serum lactate dehydrogenase (LDH) level was high (698 IU/L, normal range: 100 to 225). His alanine transaminase levels were slightly elevated (aspartate aminotransferase 50 IU/L and alanine aminotransferase 45 IU/L). His C-reactive protein level was 3.1 mg/dL, and his erythrocyte sedimentation rate was 48 mm/hour. His ferritin level was very high (1563 ng/mL, normal range: 20 to 120).

An echocardiography showed severe aortic regurgitation and a prolapsed aortic valve. A transesophageal echocardiography revealed two small vegetations. We initially considered that his symptoms resulted from congestive heart failure caused by infective endocarditis and so we began empirical therapy. However, antibiotics (ceftriaxone 2 g/day and gentamicin 180 mg/day) did not improve his symptoms or laboratory data after two weeks of therapy. A blood culture detected no bacteria.

We ordered additional laboratory tests in accordance with the guidelines for a fever of unknown origin. The results of serological tests for Epstein-Barr virus, cytomegalovirus, and mycoplasma were negative. An evaluation of connective tissue disease was nonspecific and yielded negative results for rheumatoid factor, anti-nuclear antibodies, anti-DNA antibodies, and C- and P-anti-neutrophil antibodies. Although high levels (4130 U/mL, normal range: 124 to 466) of soluble interleukin-2 receptor (sIL-2R) suggested the presence of lymphoproliferative disorders, a systemic CT scan using intravenous contrast revealed no lymphadenopathy.

A blood gas analysis showed hypoxemia and respiratory alkalosis, pH 7.461, arterial oxygen tension (PaO_2_) of 74.1 Torr, arterial carbon dioxide tension (PaCO_2_) of 27.7 Torr, and concentration of bicarbonate (HCO_3-_) of 19.5 mmol/L under room air. We performed pulmonary function tests to evaluate the cause of the hypoxemia. Our patient's forced vital capacity (FVC) and forced expiratory volume in one second (FEV_1_) were within normal limits. His total lung capacity was 5.65 L (100% predicted) and his residual volume was 2.41 L (125% predicted). His diffusion capacity (7.51 mL/minutes per mm Hg) was low (41% predicted).

**Figure 1:**
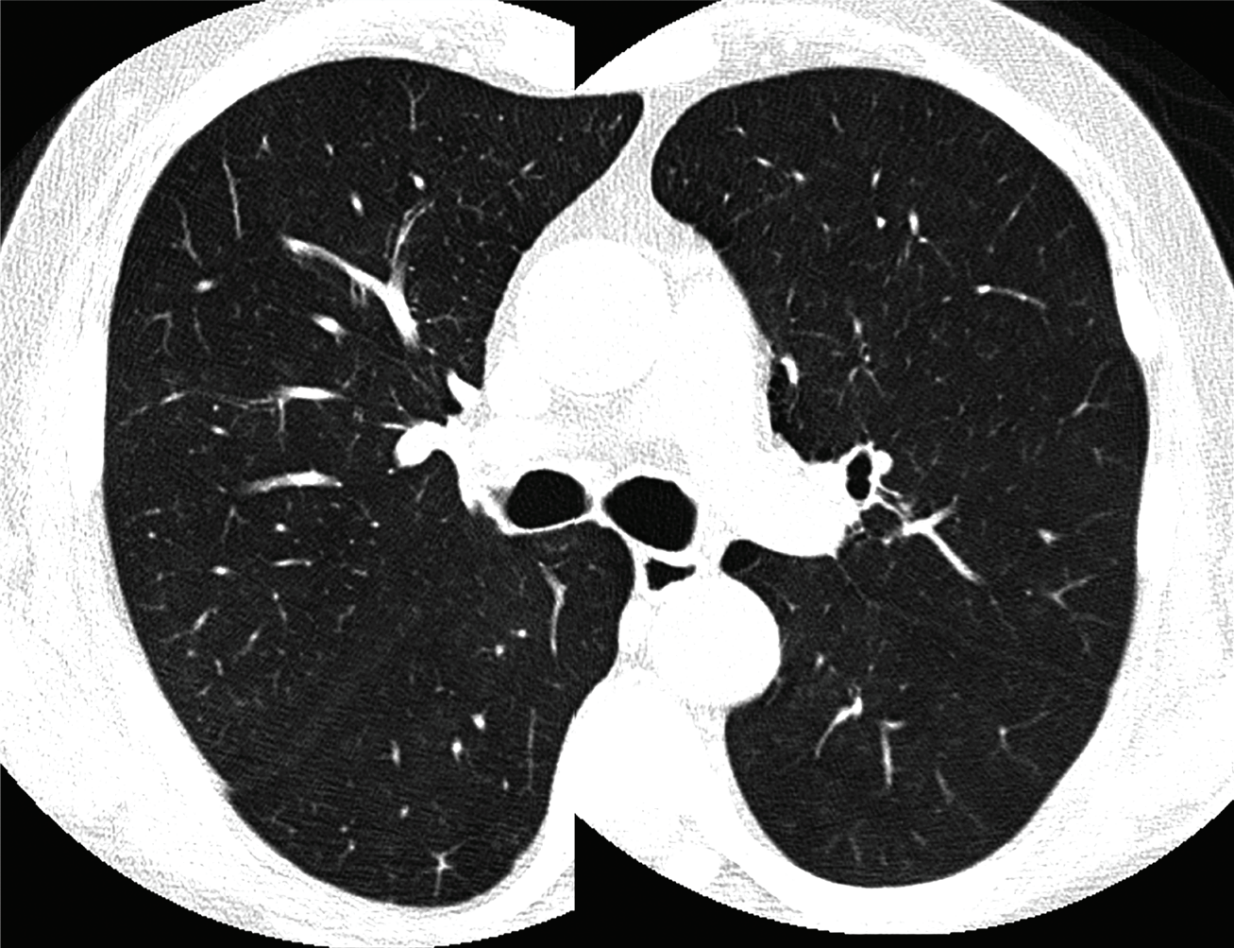
**High-resolution computed tomography image**. No structural abnormality is seen in the lung field by high-resolution computed tomography.

**Figure 2:**
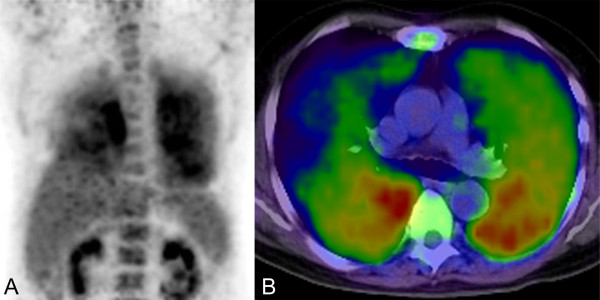
**18-Fluorodeoxyglucose positron emission tomography images**. Sagittal (A) and axial (B) images are shown. Strong tracer uptake appears in the dorsal site of the lung. The standardized uptake value in the lung field is 6.8 (120 minutes after injection).

Our patient's respiratory symptoms and decreased diffusion in the pulmonary function test indicated the necessity for further examination. However, a chest radiogram failed to demonstrate any pathological findings and an HRCT scan showed no evidence of structural abnormality in his lungs (Figure 1). Although a gallium scintigraphy showed slightly increased opacity in both lungs, it was unclear which part of his lung we should target for additional evaluations. In contrast, FDG-PET demonstrated strong increased tracer levels in the lower dorsal lung field (Figure 2). The standardized uptake values in the lung field were 5.0 at the early phase (60 minutes after injection) and 6.8 at the delayed phase (120 minutes after injection). We decided to perform TBLB from his right lower lung to explore the pathological state of his lung.

**Figure 3:**
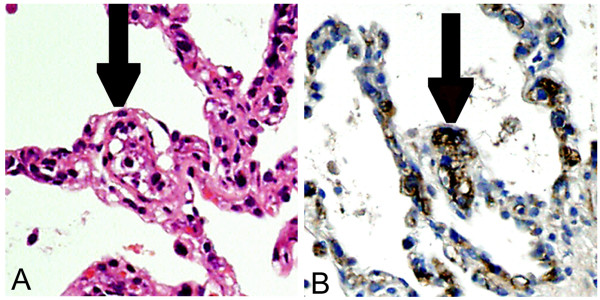
**Immunohistochemical staining**. Biopsy specimens of the lung: hematoxylin and eosin (A) and immunoperoxidase (B) stain of CD20. Black arrows show CD20^+ ^lymphocytic infiltration inside the alveolar capillary, a characteristic finding of intravascular lymphoma.

**Figure 4:**
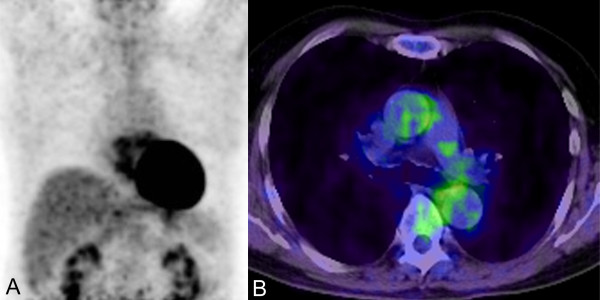
**18-Fluorodeoxyglucose positron emission tomography images after chemotherapy**. Sagittal (A) and axial (B) images are shown. Tracer uptake is not seen in the lung field six months after chemotherapy.

TBLB materials from his right lower lung revealed a diffuse atypical lymphocyte infiltration within his alveolar capillary vessels (Figure 3A), and immunohistochemical staining identified the infiltrated cells as CD20^+ ^(Figure 3B) and CD79a^+ ^(data not shown). In conclusion, FDG-PET-guided TBLB established a histological diagnosis of intravascular lymphoma. Our patient was treated with R-CHOP (rituximab, cyclophosphamide, doxorubicin, vincristine, and predonisolone) and achieved complete remission after six cycles (Figure 4). He remains alive and free of recurrent disease 24 months after chemotherapy (sIL-2R 408 U/mL and LDH 159 IU/L).

## Discussion

IVL is an aggressive and systemic disease characterized by massive proliferation of large tumor cells within the lumina of small vessels [1]. Primary pulmonary involvement is a very rare condition (approximately 5%) of IVL [2]. The diagnosis of pulmonary IVL is often difficult because of nonspecific clinical and radiographic findings, and several cases have been diagnosed during an autopsy [3,4].

FDG-PET is a noninvasive, three-dimensional, metabolic imaging technique that provides higher sensitivity than gallium scintigraphy in common lymphoma [5]. In several previous reports, TBLB was performed in patients with pulmonary IVL at an advanced stage (that is, in patients who had CT abnormalities) [6,7]. But there are only three cases (including our case) in which FDG-PET has proven useful in selecting a biopsy site for the early diagnosis of pulmonary IVL without a CT abnormality [8,9]. FDG accumulation is seen in other diffuse lung diseases such as tuberculosis, sarcoidosis, and interstitial lung diseases [10], but, owing to the rarity of pulmonary IVL, a specific pattern of tracer uptake in this disease is not well known. In our case, the pulmonary FDG uptake was diffuse, but strong accumulation was seen in the lower dorsal lung field. This accumulation pattern might reflect the gravitational effect in pulmonary circulation because distribution of pulmonary blood flow is highest in the lower lung field, "zone 3 of pulmonary circulation" [11]. IVL is a diffuse lymphoproliferative disease within small vessels; therefore, strong FDG accumulation in the lower lung field may suggest the presence of many tumor cells spreading hematogenously within pulmonary capillary vessels.

The median overall survival of IVL is 10 months, even after chemotherapy [12]. However, systemic chemotherapy at an early stage might improve the long-term prognosis of patients with IVL [13,14]. Our patient is rare in that he has pulmonary IVL and remains in complete remission more than 24 months after chemotherapy. This favorable long-term prognosis may reflect the advantage of FDG-PET, which can detect early lesions of pulmonary IVL with no CT abnormality.

## Conclusions

To the best of our knowledge, this is the first report of a long-term (24 months) survivor of pulmonary IVL diagnosed by TBLB according to FDG-PET findings. We propose that FDG-PET is a useful guide for TBLB in the early diagnosis of pulmonary intravascular lymphoma, especially in patients who have no CT abnormality.

## Abbreviations

CT: computed tomography; FDG: 18-fluorodeoxyglucose; FDG-PET: 18-fluorodeoxyglucose positron emission tomography; HRCT: high-resolution computed tomography; IVL: intravascular lymphoma; LDH: lactate dehydrogenase; R-CHOP: rituximab, cyclophosphamide, doxorubicin, vincristine, and predonisolone; sIL-2R: soluble interleukin-2 receptor; TBLB: transbronchial lung biopsy.

## Consent

Written informed consent was obtained from the patient for publication of this case report and any accompanying images. A copy of the written consent is available for review by the Editor-in-Chief of this journal.

## Competing interests

The authors declare that they have no competing interests.

## Authors' contributions

TN analyzed and interpreted the patient's data and made the initial diagnosis of intravascular lymphoma. KI and KM performed echocardiography and contributed to writing the manuscript. SK performed transbronchial lung biopsy with the assistance of HK, who interpreted the histological findings regarding intravascular lymphoma. AK and FK performed chemotherapy. KH interpreted the patient's radiological findings. MK supervised the clinical examination. FO performed the literature review and was responsible for writing the manuscript. All authors read and approved the final manuscript.
